# Sub-zero temperature biosolvent-assisted liquid–liquid microextraction for quantifying propranolol and carvedilol in human urine using HPLC-UV: greenness profile evaluation

**DOI:** 10.1039/d5ra05464h

**Published:** 2025-09-30

**Authors:** Styliani Nisyriou, Constantinos K. Zacharis

**Affiliations:** a Laboratory of Pharmaceutical Analysis, Department of Pharmacy, Aristotle University of Thessaloniki (AUTh) GR-54124 Greece czacharis@pharm.auth.gr +30 2310997663

## Abstract

Herein, a biosolvent-based liquid–liquid microextraction method utilizing menthol as the extraction medium is proposed for the isolation and quantification of selected β-blockers (propranolol and carvedilol) in human urine. The method is based on the addition of 65 μL of molten menthol to aqueous urine samples, followed by sonication. Phase separation was achieved by centrifugation, and the menthol-rich phase was subsequently solidified using sub-zero cooling. The aqueous layer was removed *via* a syringe, and the solidified menthol layer was redissolved in methanol for HPLC analysis. The key experimental parameters influencing extraction performance were optimized through a two-step multivariate approach: Plackett–Burman design (PBD) for screening and face-centered central composite design (FC-CCD) for response surface optimization. Under optimized conditions, the method exhibited linearity in the range of 50–2000 ng mL^−1^, with limits of detection of 11 ng mL^−1^ for PROP and 17 ng mL^−1^ for CARV. The method demonstrated intra-day and inter-day precision below 11%, and accuracy (% recovery) ranging from 87.2% to 110.2%. Robustness was evaluated separately for the LLME protocol and the HPLC method using Monte Carlo simulations and Plackett–Burman design, respectively. Environmental sustainability and practical applicability were assessed using the AGREEPrep, ComplexMoGAPI, Click Analytical Chemistry (CACI), and White Analytical Chemistry (WAC) metrics, confirming the method's greenness and suitability for routine analysis. Finally, the method was successfully applied to the analysis of authentic human urine samples, validating its effectiveness for real-world biological monitoring.

## Abstract 2

Herein, a biosolvent-based liquid–liquid microextraction method utilizing menthol as the extraction medium is proposed for the isolation and quantification of selected β-blockers (propranolol and carvedilol) in human urine.

## Graphical abstract

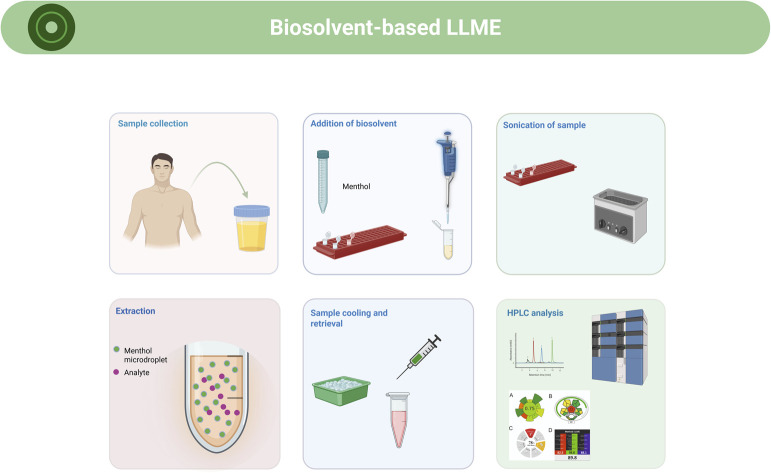

## 1. Introduction

Propranolol (PROP) and carvedilol (CARV) are beta-blockers (β-blockers), a class of medications commonly used to treat various cardiovascular conditions, including angina pectoris, atrial fibrillation, cardiac arrhythmias, and essential tremors, among others. Both drugs are listed on the World Health Organization's Essential Medicines List, recognized for their safety and effectiveness within healthcare systems. On this basis, the accurate and reliable determination of these drugs in biological fluids is vital for pharmacokinetic analysis, clinical studies, and therapeutic monitoring. Monitoring their concentration in urine is important to tailor dosage appropriately and avoid toxicity, particularly in individuals with impaired liver function or those taking other medications that influence their metabolism.^1^

Conventional extraction methods often require large volumes of solvents and lengthy procedures, leading to excessive solvent waste, prolonged analysis times, and an increased risk of contamination. In contrast, microextraction techniques represent a more efficient alternative by significantly reducing both sample and solvent consumption while enhancing extraction performance. To fully realize their potential, microextraction procedures should be designed in accordance with the principles of Green Analytical Chemistry (GAC) and Green Sample Preparation (GSP).^2,3^ GAC focuses on the development of environmentally friendly analytical methods by promoting the use of safer solvents and reagents, minimizing the disposal of toxic chemicals, enhancing researcher safety, and improving energy efficiency.^4^ Complementing this, the GSP approach specifically targets the sample preparation stage to ensure environmental sustainability and reduce risks to human health. In recent years, a variety of metric tools have been introduced including *i.e.* AGREEprep, ComplexMoGaPI, Blue Applicability Grade Index (BAGI), Whiteness Assessment using RGB 12 algorithm, “Click Analytical Chemistry Index (CACI)” and applied to evaluate the environmental impact of different analytical methodologies.^5–9^

Various strategies have been developed to enhance the sensitivity, performance, and environmental sustainability of microextraction techniques by incorporating novel nano-sorbent materials in solid-phase microextraction (SPME) and employing environmentally friendly solvents in liquid-phase microextraction (LPME).^10^ Focusing on LPME, bio-solvents are a newly introduced group of environmentally friendly solvents utilized in a range of scientific and technological applications.^11,12^ These solvents are specifically designed to exhibit reduced volatility, flammability, and toxicity, and in some cases, are more biodegradable and derived from renewable resources. As a result, they offer a substantially lower environmental and human health impact.^13^ Representative examples include monoterpenoids such as menthol, thymol, terpineol, and 1,8-cineole; monoterpene hydrocarbons like limonene; as well as other compounds such as coumarin, fatty acids, polyols, and phenols.^14^ These solvents are widely employed in analytical chemistry to mitigate environmental impact by minimizing emissions, toxicity, and potential health hazards.^15^ In recent years, these solvents have gained popularity as alternative solvents across a wide range of applications. They have also been utilized in various liquid-phase microextraction techniques, including homogeneous liquid–liquid microextraction (HLLME), deep eutectic solvent (DES)-based microextraction, and hollow fiber liquid-phase microextraction.^12^

A literature survey revealed that several sample preparation protocols have been proposed for the determination of the analytes in biological samples including DES-supported hollow fiber electromembrane extraction (HF-EME),^16^ thin-film microextraction,^17^ metal organic framework (MOF)-based packed sorbent extraction (MEPS),^18^ solvent bar microextraction (SBME),^19^ dispersive liquid liquid microextraction (DLLME),^20^ and molecularly imprinted polymer (MIP)-based stir bar sortpive extraction (SBSE).^21^ However, these methods have several disadvantages: they are time-consuming, not cost-effective, often require the fabrication of specialized sorbents, and may involve the use of toxic organic solvents. To address these limitations, environmentally friendly solvents can be employed for analyte extraction, offering improved efficiency, faster processing times, and greater ease of use.

Therefore, we have aimed at developing and validating a novel liquid–liquid microextraction (LLME) using the bio-solvent – menthol – for the isolation of PROP and CARV from human urine. Menthol is a more environmentally friendly alternative – naturally sourced, safer for human use, and easily recyclable. Due to its favorable characteristics, menthol has gained significant popularity and is widely used as an extractant for the extraction of non-polar and weak polar compounds.^22^ The key factors influencing extraction efficiency such as sample volume, menthol volume, sonication time, centrifugation speed, and duration were systematically screened and optimized using statistical experimental design approaches, including Plackett–Burman design (PBD) and face-centered central composite design (FC-CCD). The method was validated according to the FDA guidelines in terms of specificity, linearity, sensitivity, selectivity, accuracy, and precision. The robustness of the microextraction protocol and the HPLC separation were assessed using Monte-Carlo simulation experiments and PBD. The greenness of the developed method was evaluated using AGREEPrep, ComplexMoGAPI, CACI and White analytical Chemistry metric tools. The applicability of the method was demonstrated for the analysis of the analytes in authentic human urine samples.

## 2. Experimental

### 2.1. Reagents and solutions

Propranolol (PROP, >99%), carvedilol (CARV, >99%), and ethyl paraben (used as internal standard, ISTD), along with HPLC-grade methanol (MeOH), were purchased from Sigma-Aldrich (St. Louis, MO, USA). Sodium chloride (NaCl, 98%) was obtained from Honeywell/Fluka. All other reagents used were of analytical grade or higher.

Individual stock solutions of PROP, CARV, and ISTD were prepared at a concentration of 1000 μg mL^−1^ in MeOH and stored at 4 °C. Working standard solutions were freshly prepared in high-purity water daily by appropriate dilution of the stock solutions.

A Nahita 2507/15 microcentrifuge and an Elmasonic Easy 30H ultrasonic bath (Elma Schmidbauer GmbH, Germany) were used for sample processing. High-purity water was obtained using a B30 water purification system (Adrona SIA, Riga, Latvia). Cellulose acetate membrane filters were procured from ISOLAB Laborgeräte GmbH (Eschau, Germany).

### 2.2. HPLC conditions

HPLC analyses were carried out using a Shimadzu 2010A HPLC-UV system (Kyoto, Japan), consisting of a quaternary pump, thermostated autosampler, column oven, and dual-wavelength UV detector. Instrument operation and data acquisition were managed using LC Solutions software (version 1.24 SP4).

Chromatographic separation of the target drugs was performed on a Discovery® HS C_18_ column (150 × 4.6 mm, 5 μm; Supelco), maintained at 25 °C. The mobile phase comprised 0.1% formic acid in water and methanol (50 : 50, v/v), delivered at a flow rate of 1.0 mL min^−1^. The injection volume was 10 μL, and UV detection was conducted at 230 nm.

### 2.3. Sample collection and handling

Urine samples were collected from healthy adult volunteers using sterile collection cups, following the provision of written informed consent. All participants were fully informed about the study procedures, and no medications were administered prior to sample collection. The samples were immediately stored at −18 °C until further analysis.

### 2.4. LLME protocol

**Fig. 1:**
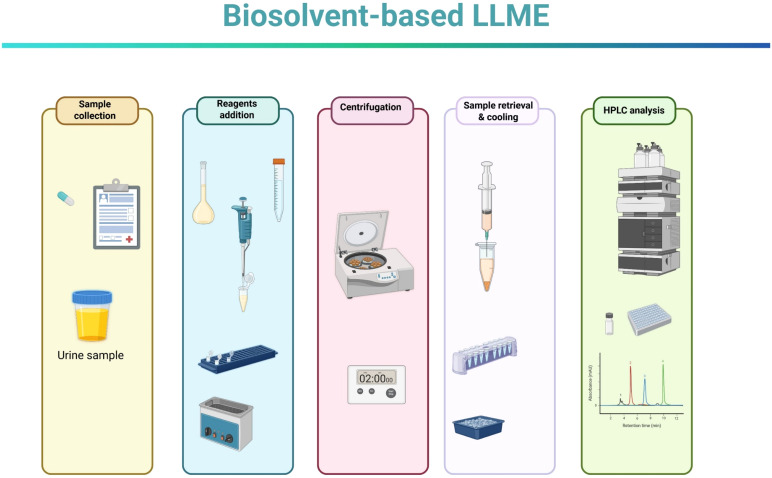
Schematic presentation of the main analysis steps. Created in BioRender. Zacharis, C. (2025) https://BioRender.com/ut6x9gu.

The main steps of the LLME procedure are illustrated in Fig. 1. Briefly, 250 μL of undiluted human urine was mixed with 150 μL of NaCl solution (30% w/w), 50 μL of ISTD solution, and 50 μL of either analyte mixture or water (for blank samples). Then, 65 μL of molten menthol (preheated to 40 °C) was added as the extraction solvent. The mixture was vortexed for 10 seconds and sonicated for 30 seconds to promote the dispersion of menthol microdroplets within the aqueous phase. Following dispersion, the sample was centrifuged at 10k rpm for 2 min and immediately placed in an ice bath to accelerate solidification of the menthol phase. The aqueous layer was carefully removed and discarded using a disposable syringe. The solidified menthol phase, enriched with analytes, was then dissolved in 500 μL of MeOH. The final solution was transferred to an HPLC vial for analysis.

## 3. Results and discussion

A multivariate optimization strategy was employed to refine the LLME parameters. This approach aligns with the principles of Green Analytical Chemistry by substantially minimizing the number of experimental runs, along with the consumption of samples, reagents, energy, and time.^2^ A two-step multivariate optimization approach was employed to refine experimental parameters. Initially, a PBD was used as a screening tool to identify statistically significant factors – excluding the type of bio-based solvent – from those with negligible effects. Subsequently, an FC-CCD was implemented to determine the optimal levels of the identified significant variables.

### 3.1. Study of bio-solvent type

The LLME procedure involves adding a defined volume of extraction solvent to the sample solution, enabling the partitioning of the analyte between the two phases. The efficiency of analyte extraction is mostly influenced by the type of solvent. Preliminary experiments employed 1 mL of aqueous solution mixed with 50 μL of molten bio-based solvent, which had been preheated in a 60 °C water bath. Dispersion of the solvent in the aqueous layer was facilitated using sonication. The mixture was vortexed and centrifuged to promote phase separation. Following this, the system was cooled to 4 °C to solidify the bio-based solvent layer, enabling easy collection. The aqueous phase was removed using a syringe, and the solidified menthol layer was dissolved in MeOH prior to HPLC analysis.

Three bio-based monoterpene solvents—menthol, terpineol, and thymol—were evaluated for use as extraction media. These solvents are inexpensive, non-toxic, and possess melting points near ambient temperature. Due to their lower densities compared to water, they remain on the surface of the aqueous phase during extraction, facilitating easy removal using a spatula. Thymol yielded higher extraction recoveries of the analytes (51–58%) compared to terpineol (36–42%) and menthol (23–29), respectively. However, significant variability (>30%) was observed, likely due to the relatively high viscosity of molten thymol. To address this issue, extractions were performed using various methanolic mixtures (thymol/CH_3_OH: 25/75, 50/50, and 75/25 v/v) in which no solidification occurred. Despite these efforts, the challenges associated with thymol ultimately prevented its use as extraction solvent. Terpineol did not completely solidify under certain experimental conditions, likely due to their relatively high solubility in water (7100 mg L^−1^ at 20 °C (ref. 23)). As a result, complete phase separation between the bio-based solvent could not be achieved. Analogous findings were observed in our previous research work.^24^ As a result, menthol was chosen for subsequent experiments.

### 3.2. Screening of LLME parameters using PBD

**Fig. 2:**
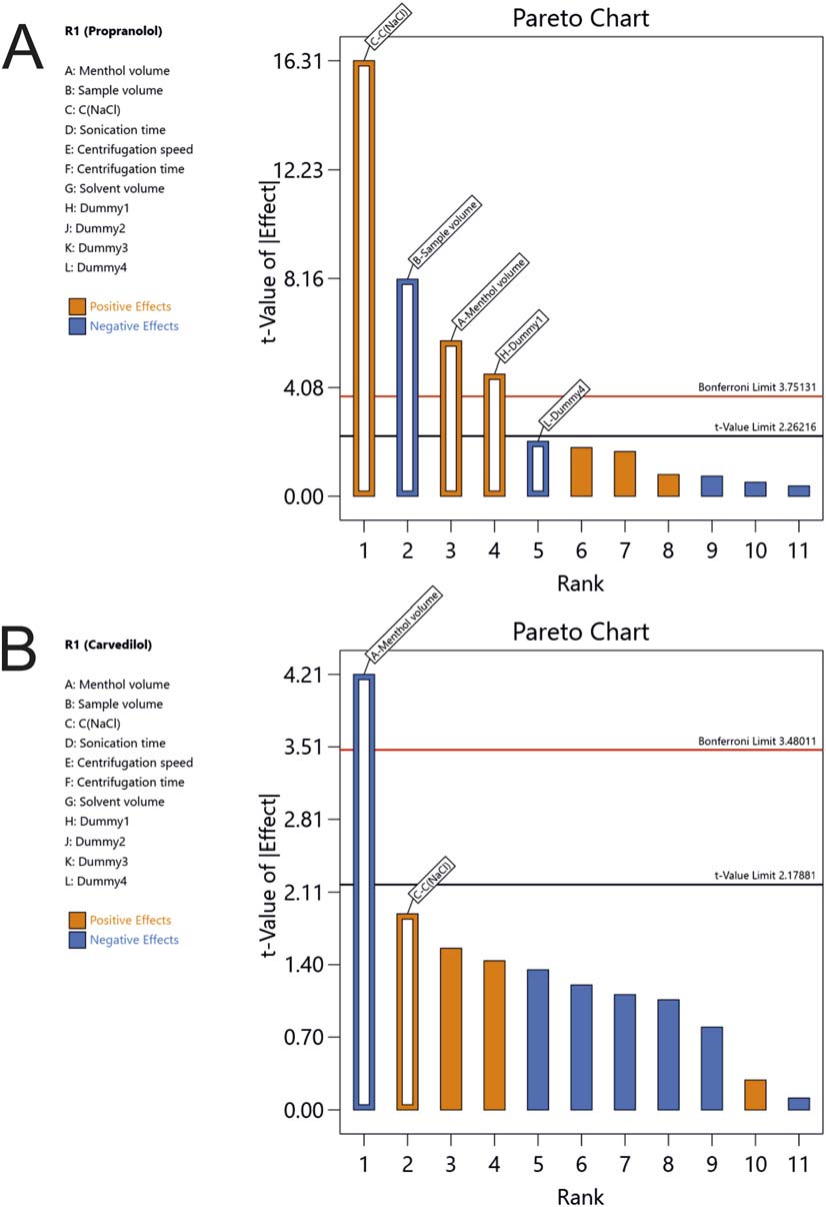
PBD for the screening of LLME parameters for (A) propranolol and (B) carvedilol.

A cost-effective Plackett–Burman design (PBD) was constructed to screen seven experimental factors—menthol volume (A), sample volume (B), NaCl concentration (C), sonication time (D), centrifugation speed (E), centrifugation time (F), and dissolution solvent volume (G) – along with four dummy variables (Dummy1–Dummy4) to estimate experimental error. The design required 15 experimental runs, which were performed in randomized order to minimize systematic bias. The tested factors, their corresponding levels, and the experimental matrix are presented in Tables S1 and S2. Extraction recovery (%ER) was selected as the response variable for the optimization process. Fig. 2 presents the Pareto ranking plots obtained from multivariate regression analysis, illustrating the influence of the investigated factors. The bar lengths correspond to the absolute values of the standardized effects, while the vertical black line indicates the threshold for statistical significance at the 95% confidence level.

Menthol volume was identified as a statistically significant factor influencing the %ER of both analytes. In contrast, NaCl concentration and sample volume were significant (*p* < 0.05) only for PROP, while the remaining variables showed no notable effects. The findings indicate that PROP extraction efficiency increased at lower sample volumes (negative effect) and under high salinity conditions (positive effect). This enhancement is likely due to the salting-out phenomenon, wherein elevated NaCl concentrations reduce the analyte's solubility in the aqueous phase, thereby promoting its transfer into the menthol microdroplets. Furthermore, higher NaCl concentrations led to an increase in the volume of solidified menthol, likely due to the reduced solubility of menthol in saline solutions. Similar salting-out behavior has been reported for β-blockers in other microextraction techniques.^25,26^

Based on these findings, factors A (menthol volume), B (sample volume), and C (NaCl concentration) were selected for further optimization using a FC-CCD. The non-significant parameters were fixed at values aimed at minimizing solvent consumption and analysis time (sonication time: 30 s, centrifugation speed/time: 10 000 rpm for 2 min, dissolution solvent volume: 500 μL).

### 3.3. Optimization of LLME parameters using FC-CCD

A FC-CCD was employed to optimize menthol volume, sample volume, and NaCl concentration. The design consisted of a full factorial design at two levels (2^3^ = 8 experiments), augmented with axial (star) points (2 × 3 = 6 experiments) to achieve desirable properties such as rotatability and orthogonality, facilitating the fitting of a second order (quadratic) polynomial model. Additionally, six center points were incorporated to assess experimental variability and enable the estimation of lack of fit (LOF). The tested factors, their coded and actual levels, and the experimental matrix are detailed in Table 1. To minimize systematic bias, all experiments were conducted in a randomized order. A second-order polynomial model was fitted to the experimental data using multivariate regression analysis as follows:
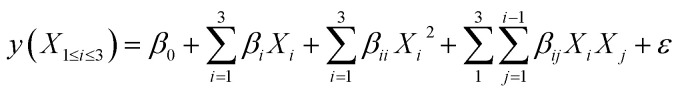
where *y*(*X*_1≤*i*≤3_) indicates the experimental response, *X*_*i*_ the factor, *β*_0_ the constant, *β*_*i*_ the linear coefficients, *β*_*ii*_ the quadratic coefficients, *β*_*ij*_ the quadratic coefficients and *ε* the random error. High-order models (*e.g.* cubic model) were aliased for all ER suggesting augmentation of the design.

**Table 1:** Experimental FC-CCD domain for the optimization of LLME parameters

Standard run	Menthol volume (μL)	Sample volume (μL)	NaCl concentration (% w/v)	%ER	
				PROP	CARV
15	137.5	750	10	47.06	72
8	250	1000	20	53.17	59
11	137.5	500	10	65.52	73.6
12	137.5	1000	10	33.8	66.5
5	25	500	20	57.2	79.4
16	137.5	750	10	48.88	76.1
9	25	750	10	17.81	75.4
4	250	1000	0	4.94	59
17	137.5	750	10	50.7	77.9
1	25	500	0	6.5	69.6
13	137.5	750	0	5.33	67.3
20	137.5	750	10	46.67	73.6
6	250	500	20	85.15	71.6
3	25	1000	0	5.07	44.8
14	137.5	750	20	68.77	78.3
10	250	750	10	51.35	65.4
18	137.5	750	10	52.26	76
19	137.5	750	10	34.84	73.8
7	25	1000	20	34.06	51.9
2	250	500	0	9.36	63.1

The experimental design and statistical analyses were conducted using Design Expert® version 13 (Stat-Ease Inc., Minneapolis, MN, USA). Analysis of variance (ANOVA) was used to identify significant main effects and interactions, with results summarized in Tables S3 and S4 (SI Material). The observed synergistic effects of the studied parameters can be attributed to (i) enhanced analytes transfer *via* salting-out, (ii) improved phase separation due to reduced menthol solubility, and (iii) optimized solvent-to-sample ratio that maximizes enrichment efficiency. The lack-of-fit (LOF) *p*-values exceeded 0.05, indicating that the LOF was not statistically significant and that the models were suitably fitted relative to the pure error. The coefficient of determination (*R*^2^) values exceeded 0.8927, and the adjusted *R*^2^ values were greater than 0.8432, confirming the model's robustness and its ability to explain the observed variance in the response. Adequate precision values, which measure the signal-to-noise ratio, were above 14.47 in all cases, further supporting the models' reliability.

**Fig. 3:**
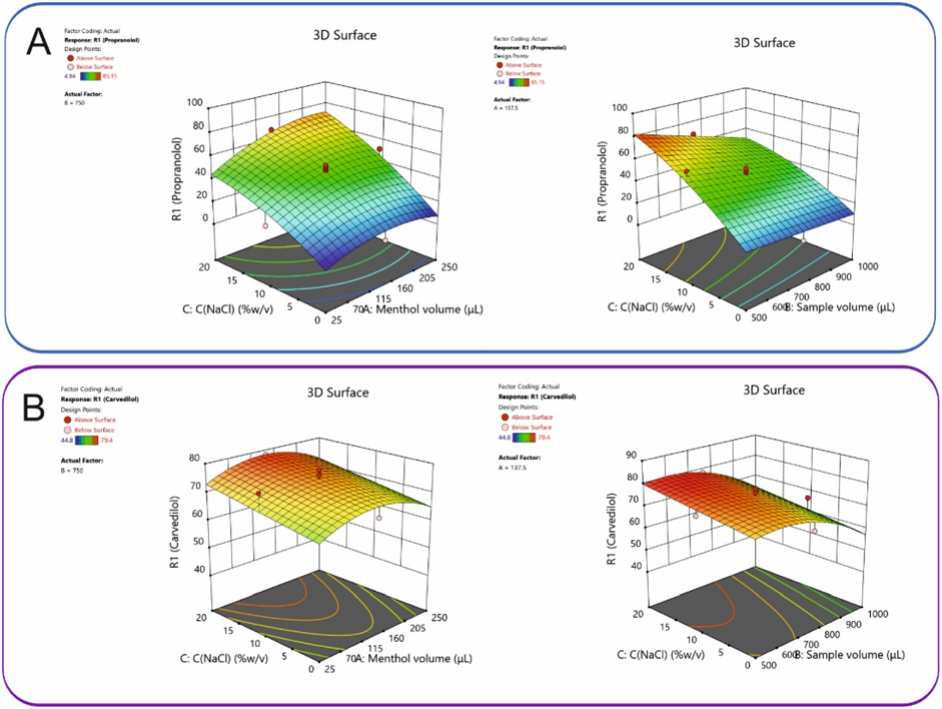
3D plots of the effect of NaCl concentration, menthol and sample volumes on the %ER of (A) PROP and (B) CARV.

Model validity was additionally confirmed through residual diagnostics: the normal probability plot of residuals and the residuals *versus* predicted values plot demonstrated a random distribution, indicating that the assumptions of normality and homoscedasticity were met (Fig. S1, SI Material). The 3D response surface plots for all analytes, presented in Fig. 3, illustrate the synergistic effects of menthol volume, NaCl concentration, and sample volume, which collectively yielded a clear optimum in extraction efficiency. Derringer's desirability function (*D*) was employed to identify the optimal experimental conditions. This function provides a scale ranging from 0 (completely undesirable) to 1 (fully desirable), with higher values indicating better overall optimization. As shown in the desirability surface contour plots (Fig. S2), a composite desirability value of 0.669 was obtained. The corresponding optimum conditions were determined to be 65.9 μL of menthol volume, 9.05% w/v NaCl concentration, and 500 μL of sample volume. For practical implementation, the menthol volume and NaCl concentration were rounded to 65 μL and 9% w/v, respectively. To verify the robustness of these optimized conditions, six replicate extractions were performed. The deviation between predicted and experimental values was found to be less than 7%, confirming the reliability and accuracy of the optimization process.

### 3.4. Study of cooling conditions

The solidification of the menthol layer is a critical step in minimizing the overall duration of the LLME procedure. Preliminary experiments showed that approximately 10 min at 4 °C were required for menthol to solidify, likely due to the poor thermal conductivity of plastic Eppendorf tubes. To expedite the process, sub-zero temperatures were employed using an ice bath, which enabled menthol solidification in less than 1 minute. This approach also allowed for the simultaneous processing of multiple samples, significantly increasing sample throughput. However, extending the freezing time to 10 minutes resulted in solidification of the aqueous phase as well, hindering its recovery *via* syringe. Therefore, a 2 min cooling period was selected as optimal. Utilizing an ice bath, phase separation of several samples was efficiently achieved, substantially reducing average processing time per sample and enhancing the overall analytical workflow.

### 3.5. Method validation

The proposed analytical method was validated for key parameters, including linearity, precision, accuracy, selectivity, limit of detection (LOD), limit of quantitation (LOQ), and robustness.

Selectivity should be evaluated to demonstrate the ability of the bioanalytical method to accurately measure and distinguish the analytes in the presence of other components – whether endogenous or exogenous – within the sample matrix under certain analytical conditions. In methods with inherently low selectivity (*e.g.*, HPLC with non-MS detection), selectivity is not solely dependent on the analytical step but also significantly influenced by the initial sample clean-up process.

**Fig. 4:**
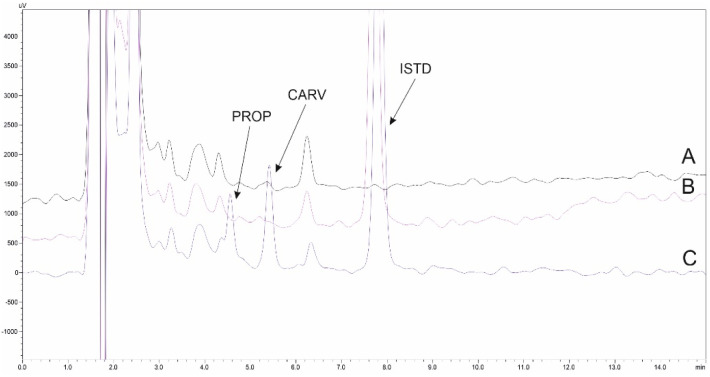
Representative HPLC-UV chromatograms of the analysis of pooled urine sample (*n* = 6) (A) unspiked, (B) spiked with ISTD and (C) spiked with drugs (at 500 ng mL^−1^) and ISTD.

In this study, selectivity was evaluated by analyzing a drug-free pooled human urine sample (*n* = 6). Representative HPLC-UV chromatograms of a blank sample and a spiked sample containing the ISTD and the analytes are shown in Fig. 4. As illustrated, no interfering peaks were observed in the blank urine sample at the retention times of PROP and CARV. This confirms that the optimized sample preparation procedure effectively removes matrix components, ensuring selective detection of the target analytes.

To assess linearity, calibration curves were constructed for each drug in both aqueous and matrix-matched solutions, using independently extracted samples at each concentration level. The peak area of each analyte against ISTD *versus* the concentration were plotted. The results obtained are summarized in Table 2. The slope ratio of aqueous and matrix-matched calibration curves was found to be 2.1 and 1.5 for PROP and CARV, respectively, exceeding the acceptable range for matrix effect evaluation (0.8–1.2). These findings indicate significant matrix effects, rendering external calibration unsuitable. As a result, matrix-matched calibration curves were employed for accurate quantification of the analytes in real urine samples. The limits of detection (LOD) and quantification (LOQ) were determined based on signal-to-noise (S/N) ratios of 3 and 10, respectively. The LLOQ was defined as the lowest analyte concentration on the calibration curve that could be quantified with a %RSD ≤ 20% and accuracy within the range of 80–120%.

**Table 2:** Linearity, LOD, LOQ, LLOQ data of the developed method for the analytes

Parameter	PROP	CARV
Linear range (ng mL^−1^)	100–2000	50–2000
Slope_aqueous_ ± SD	(66 ± 1.8) × 10^−6^	(212 ± 8) × 10^−6^
Intercept_aqueous_ ± SD	(50 ± 240) × 10^−6^	(−125 ± 749) × 10^−5^
Coefficient of determination (*r*_aqueous_)	0.9985^a^	0.9962^b^
Slope_matrix-matched_ ± SD	(139 ± 4) × 10^−6^	(326 ± 12) × 10^−6^
Intercept_matrix-matched_ ± SD	(−309 ± 351) × 10^−5^	(405 ± 1087) × 10^−5^
Coefficient of determination (*r*_matrix-matched_)	0.9978^a^	0.9967^b^
LOD (ng mL^−1^)	17	11
LOQ (ng mL^−1^)	56	36
LLOQ (ng mL^−1^)	100	50

a Number of data points: 6.

b Number of data points: 7.

The method's accuracy and precision were evaluated by conducting three replicate extractions (*n* = 3) within a single day to assess intra-day variability, and by performing triplicate measurements over three consecutive days to assess inter-day variability. Based on this, spiked drug-free human urine samples at three concentration levels – low (LQC), medium (MQC), and high (HQC) – were analyzed using the developed method. For both analytes, the intra-day precision (%RSD) was below 11.0%, while inter-day %RSD values were under 9.0% (Table S5). Additionally, the relative recovery (%RR) ranged from 87.2% to 110.2% across all quality control levels, confirming the method's reliability with respect to both precision and accuracy.

The robustness of the microextraction protocol was rigorously evaluated through Monte Carlo simulation and process capability analysis using Minitab Workspace 1.5.1.0 (trial version). A total of 100k iterations were conducted, with the resulting simulated datasets used to calculate process capability indices (*C*_p_*k*). The acceptance criterion for %ER was set within ±10% of the predicted value established during the optimization phase. The capability analysis demonstrated that *C*_p_*k* values exceeded 1.33 when the SD values for menthol volume, sample volume, and NaCl concentration were 1 μL, 15 μL, and 0.25% (w/v), respectively. A representative histogram of the capability analysis results is presented in Fig. S3. The robustness of the HPLC separation method was systematically evaluated using a PBD. Eight factorial experiments were constructed using TIBCO Statistica 13.3.0 (TIBCO Software Inc., Palo Alto, CA, USA) to assess the main effects of selected variables on the resolution between the two analytes and the ISTD, as summarized in Table S6. The Pareto chart (Fig. S4) indicated that the %MeOH content in the mobile phase and the column temperature had the most significant impact on resolution. In contrast, flow rate and formic acid concentration exerted relatively minor effects. Despite these variations, the lowest resolution value (*R*_1_) recorded was 2.54, which complies with the minimum resolution criteria established by the FDA's Center for Drug Evaluation and Research (CDER).^27^

**Fig. 5:**
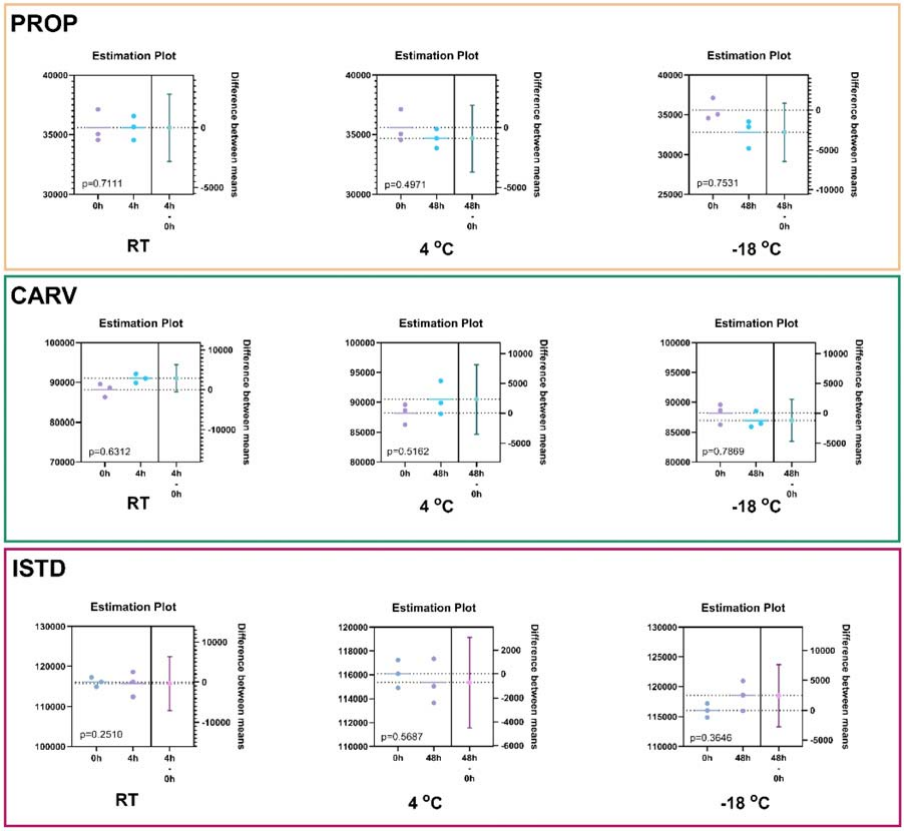
Stability study of the drugs and ISTD in unprocessed blank urine sample. Numerical values obtained from Welch’ test and represent the *p*-values of the comparison of the peak area at each time span with the control sample (*t* = 0 h).

The stability of drugs during sample processing and storage is a critical factor in clinical chemistry, as it ensures the accurate interpretation of measured drug concentrations. Therefore, the stability of the analytes and ISTD in the urine matrix was assessed under three storage conditions: (i) at room temperature for up to 4 h, (ii) at +4 °C for up to 48 h, and (iii) at −18 °C for up to 48 h. Following each storage period, samples were pretreated according to the procedure described in Section 2.4. Using the Welch's test (95% CI) (Fig. 5), no statistically significant degradation of either analytes or ISTD was observed under any of the tested conditions. These results align with stability data previously reported in the literature.^20^

### 3.6. Application to human urine samples

To evaluate the practical applicability of the developed method, several individual human urine samples were analyzed. The authentic samples were spiked with known concentrations of the target analytes at three levels (250–2000 ng mL^−1^) and subjected to the optimized extraction and analysis protocol. The relative recoveries obtained ranged from 89.3% to 116.7%, and the corresponding %RSD values were all below 13.1%, confirming the method's reliability and suitability for real-world biological sample analysis.

### 3.7. Comparison with other published approaches

The performance of the proposed microextraction method was benchmarked against several previously reported techniques for analyte determination in biological matrices, including graphene oxide-based MIP (GO-MIP),^21^ chitosan@MOF-199-based MEPS,^18^ DES-supported HF-EME,^16^ DLLME,^20^ and FPSE.^28^ Unlike these methods, the developed LLME protocol does not require solid-phase materials such as membranes, sorbents, or cartridges, thus offering a more economical and simplified approach. Furthermore, the extraction process is completed in just 5 min, which is considerably faster than GO-MIP (40 min), MEPS (24 min), and HF-EME (20 min), thereby enhancing sample throughput. In addition to its speed and simplicity, the method also demonstrated excellent sensitivity, precision, and accuracy, as detailed in Table 3.

**Table 3:** Comparison of the proposed method with previously published bioanalytical HPLC approaches

Sample	Sample pretreatment^a^	Extraction time (min)	LOQ (ng mL^−1^)	RSD (%)	RR (%)	Method's whiteness	Ref.
Urine	GO-based MIP	40	1	<7.3 (intra-day)	86.8–106.3	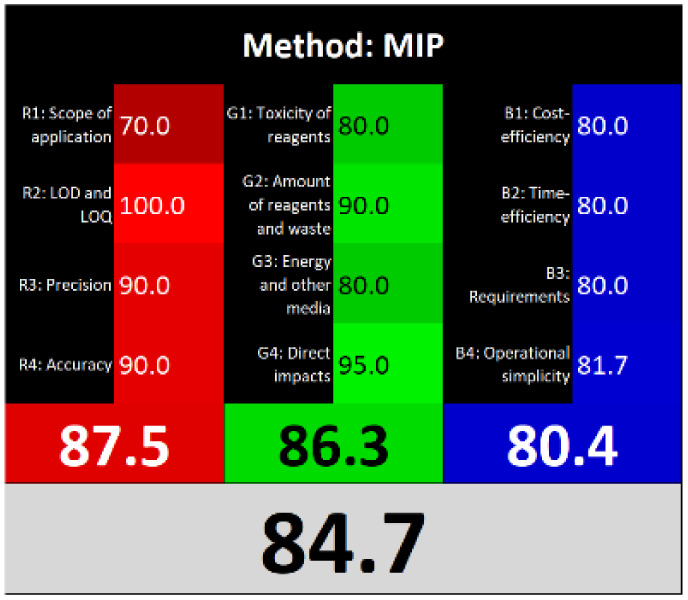	21
Saliva, plasma, urine	Chitosan@MOF-199-based MEPS	24	5	<4.7 (intra-day)	87.2–112.2	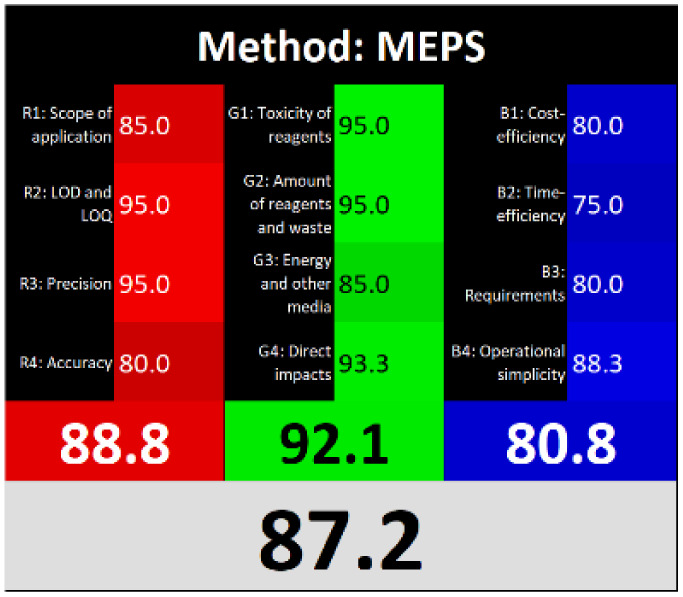	18
				<8.1 (inter-day)			
Urine, plasma	DES-supported HF-EME	20	0.75	<4.5 (intra-day)	94.6–108.6	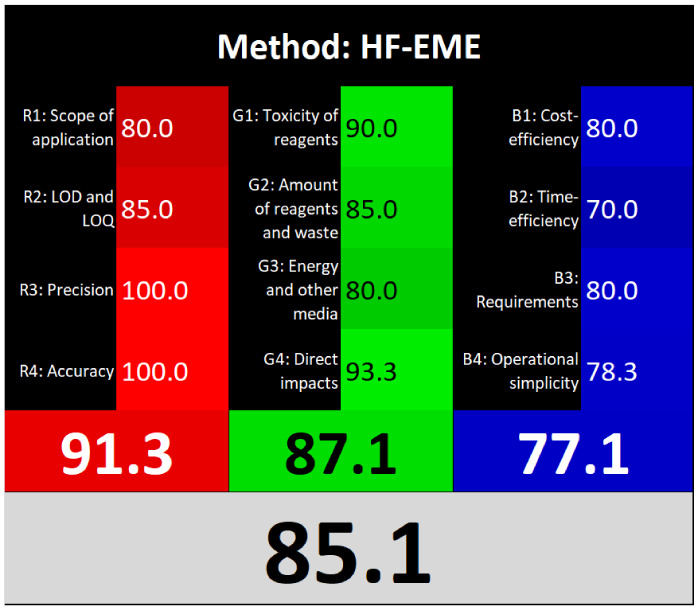	16
				<6.2 (inter-day)			
Plasma	DLLME	12	20	<9.7 (intra-day)	−14.4–3.8 (as % bias)	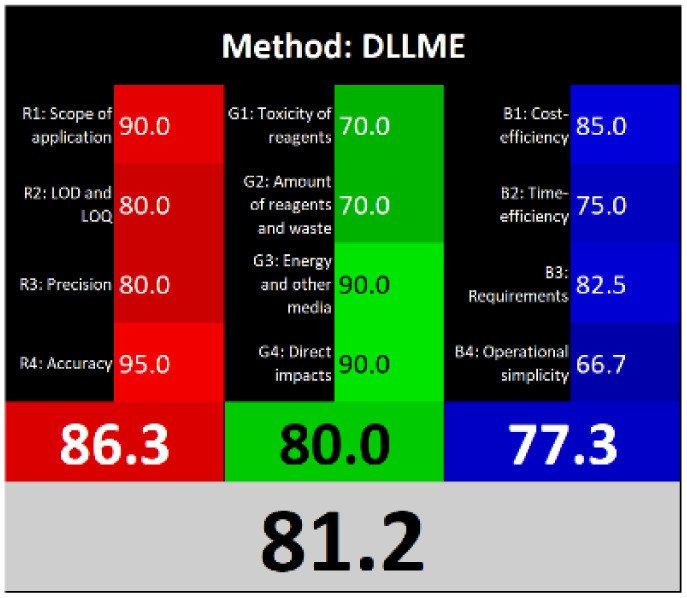	20
				<10.4 (inter-day)			
Urine, serum	Sol–gel Carbowax 20 M-based FPSE	15	50	<11.5 (intra-day)	−17.2–13.3 (as % bias)	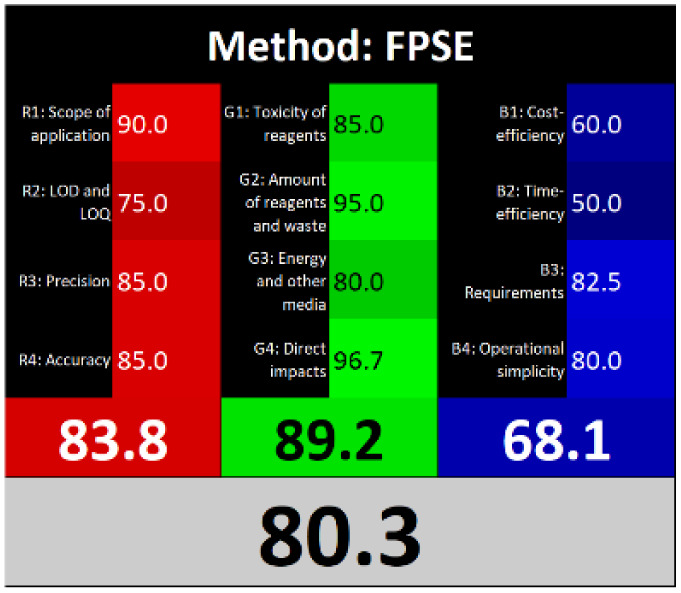	28
				<14.5% (inter-day)			
Urine	Biosolvent-based LLME	5	50	<11.0 (intra-day)	87.2–107.5	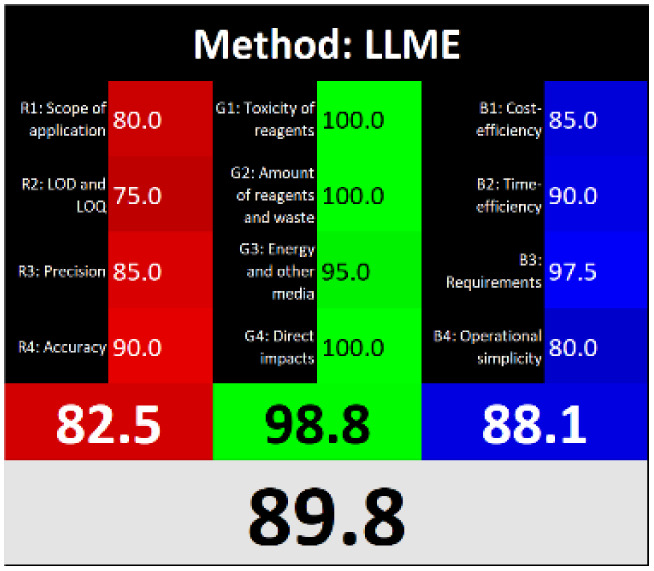	Proposed method
				<9.0 (inter-day)			

a GO-based MIP: graphene oxide-based molecularly imprinted polymer; MEPS: microextraction by packed sorbent; DES-supported HF-EME: Deep eutectic solvent-supported hollow fiber-electromembrane extraction; DLLME: dispersive liquid liquid microextraction; FPSE: Fabric-phase sorptive extraction.

### 3.8. Sustainability assessment

Various assessment tools have been developed to evaluate the alignment of analytical methods with the principles of GAC.^29^

AGREEprep is a specialized metric developed to evaluate the environmental sustainability of sample preparation procedures. It generates a circular pictogram featuring a central numerical score ranging from 0 (indicating poor environmental performance) to 1 (indicating optimal sustainability or no sample preparation required).^5^ The color and value of the central circle reflect the overall greenness of the sample preparation approach. Surrounding the center, ten individual segments represent specific evaluation criteria. Each segment's length corresponds to the default weight of its respective criterion, while its color visually communicates the level of compliance with environmentally friendly practices.

ComplexMoGAPI is an enhanced version of the ComplexGAPI tool, designed to provide a more comprehensive evaluation of the environmental impact of analytical methods. It integrates visual assessment with a quantitative scoring system, offering detailed scores on a scale from 0 to 100 to reflect the overall greenness of a method.^6^

The “Click Analytical Chemistry Index” (CACI) is a recently developed tool designed to evaluate the practicality and efficiency of analytical methods, inspired by the principles of click chemistry for their simplicity, robustness, and reliability.^30^ This assessment model considers several critical aspects, including sample volume, preparation complexity, method feasibility, applicability, portability, sensitivity, and potential for automation. Each criterion is scored, and the method's overall performance is visually summarized using a color-coded pictogram: colored segments indicate high performance, gray reflects moderate performance, and black signifies poor performance or non-adherence to the specified criteria.

Recently, the concept of ‘White Analytical Chemistry’ (WAC) has emerged as an extension of Green Analytical Chemistry (GAC), aiming to evaluate the overall sustainability of analytical methods by integrating environmental, economic, and analytical performance aspects.^31^ The WAC framework proposes a color-coded model that consolidates the 12 principles of GAC into a more holistic sustainability assessment. It categorizes these principles into three core dimensions: ‘green’ (G1–G4), addressing chemical safety, reduction in sample and solvent usage, waste minimization, and energy efficiency; ‘red’ (R1–R4), focusing on analytical performance parameters such as applicability, sensitivity, precision, and accuracy; and ‘blue’ (B1–B4), which emphasize economic and operational factors, including cost-effectiveness, time efficiency, and procedural simplicity.

**Fig. 6:**
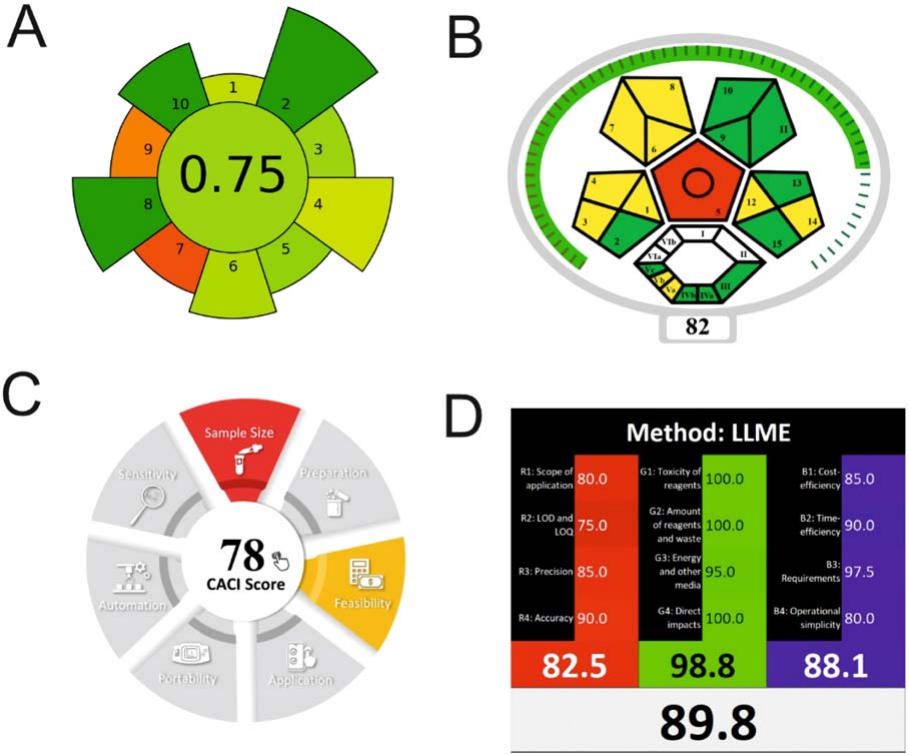
(A) AGREEprep, (B) ComplexMoGAPI, (C) CACI and (D) WAC scores for the developed method for the determination of PROP and CARV.

Τhe overall score of AGREEprep assessment was found to be 0.75 (Fig. 6A) with further details provided in Fig. S5. Notably, criterion 2 scored well due to the absence of highly toxic reagents. However, only two criteria were below 0.5: integration and automation (criterion 7), and post-sample preparation configuration (criterion 9). Using the ComplexMoGAPI tool, the method achieved a cumulative greenness score of 82, reflecting a relatively high level of environmental sustainability (Fig. 6B). More details are provided in Fig. S6. The estimated CACI score of the proposed is 78, which is acceptable in terms of practicality. As a result, the developed analytical method exhibits a level of environmental sustainability when assessed through a green evaluation (Fig. 6C). Finally, the RGB12 algorithm resulted in good whiteness value of 89.8 higher than other bioanalytical microextraction-based methodologies (Fig. 6D).

The RGB12 algorithm was employed to evaluate the overall 'whiteness' of the developed procedure in comparison to recently published approaches. As presented in Table 3, the proposed method achieved a high whiteness score of 89.8. This favorable outcome reflects the intentional implementation of strategies aimed at reducing energy consumption, minimizing reagent use, and decreasing waste production. These results highlight the method's enhanced sustainability, operational efficiency, and cost-effectiveness, positioning it as a superior alternative for routine bioanalytical applications.

## 4. Conclusions

A bio-based liquid–liquid microextraction (LLME) method employing menthol as the extraction solvent was developed for the determination of propranolol and carvedilol in human urine. This approach is environmentally friendly, straightforward, and time-efficient, requiring only minimal solvent consumption. Key extraction parameters were systematically optimized using design of experiments, including screening and response surface methodologies. The method exhibited satisfactory linearity, precision, and accuracy under optimized conditions. Notably, the use of just 65 μL of menthol underscores the method's alignment with green analytical chemistry principles. Furthermore, the entire analytical procedure is conducted under ambient conditions, reinforcing its practicality for routine use in standard laboratory settings. Overall, the proposed method offers a reliable, sustainable, and effective strategy for the quantification of the certain β-blockers in biological matrices.

## Author contributions

Styliani Nisyriou: Investigation, formal analysis, data curation, validation, writing – original draft, Constantinos K. Zacharis: Conceptualization, investigation, methodology, supervision, visualization, writing – original draft, writing – review & editing.

## Conflicts of interest

There are no conflicts to declare.

## Supplementary Material

RA-015-D5RA05464H-s001

## Data Availability

The data supporting (DoE raw data, Monte-Carlo simulation graph, Pareto chart, input parameters for sustainability assessment tools, ANOVA table) of this article have been included as part of the SI. Supplementary information is available. See DOI: https://doi.org/10.1039/d5ra05464h.
