# Supplementary material for: Sub-zero temperature biosolvent-assisted liquid–liquid microextraction for quantifying propranolol and carvedilol in human urine using HPLC-UV: greenness profile evaluation
Source: RSC Adv. 2025 Sep 30;15(43):36093–102. doi: 10.1039/d5ra05464h (PMC12481188; doi:10.1039/d5ra05464h)
Supplement: RA-015-D5RA05464H-s001 [file RA-015-D5RA05464H-s001.pdf]

Supplementary Information (SI) for RSC Advances.
This journal is © The Royal Society of Chemistry 2025

Supplementary information

Sub-zero Temperature Biosolvent-Assisted Liquid–Liquid Microextraction for Quantifying Propranolol and Carvedilol in Human Urine Using HPLC-UV: Greenness Profile Evaluation

Styliani Nisyriou, Constantinos K. Zacharis*

Laboratory of Pharmaceutical Analysis, Department of Pharmacy, Aristotle University of Thessaloniki, GR-54124, Greece

*Corresponding author
Constantinos K. Zacharis
Associate Professor
Laboratory of Pharmaceutical Analysis, School of Pharmacy,
Aristotle University of Thessaloniki (AUTh),
GR-54124, Greece
Tel: +30 2310997663
E-mail: czacharis@pharm.auth.gr

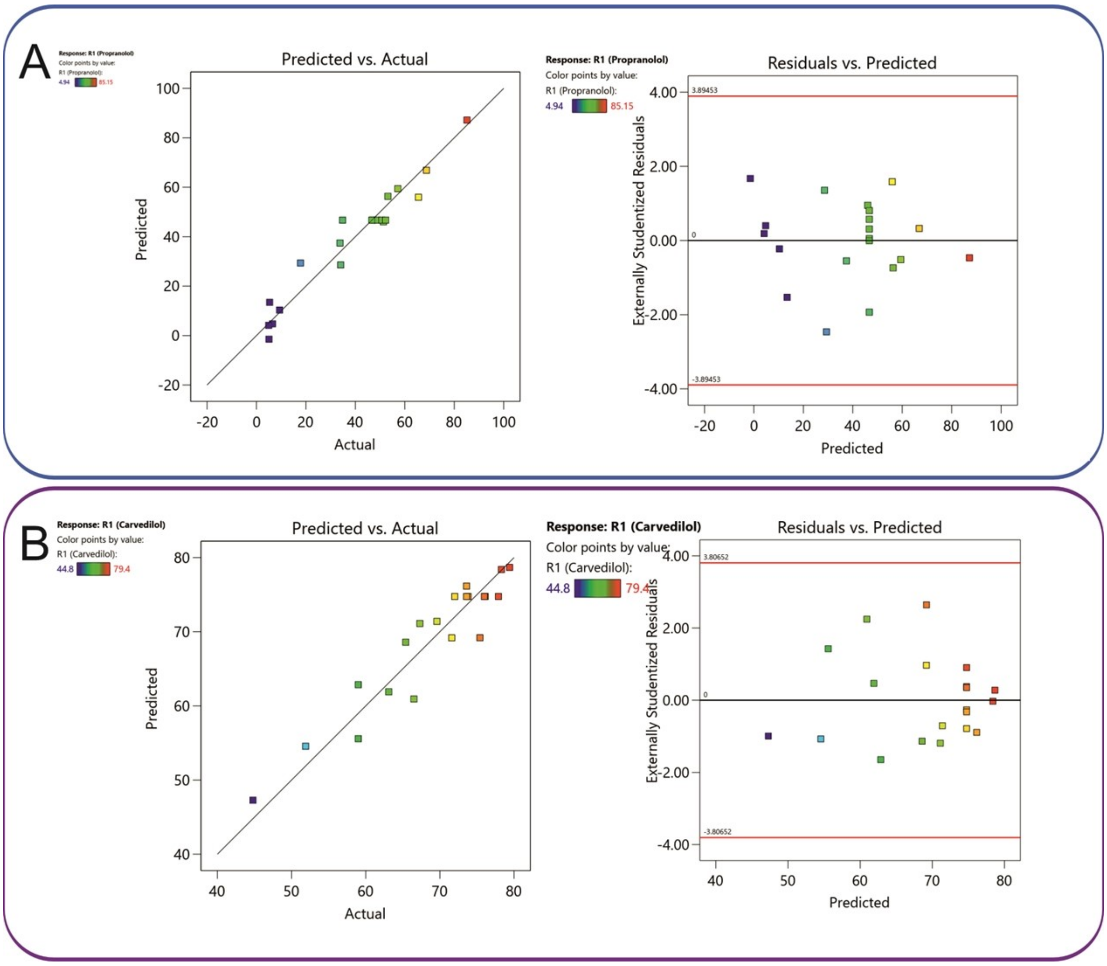

Figure S1. Normal probability and the residuals vs predicted plots for the %ER of A) PROP, and B) CARV.

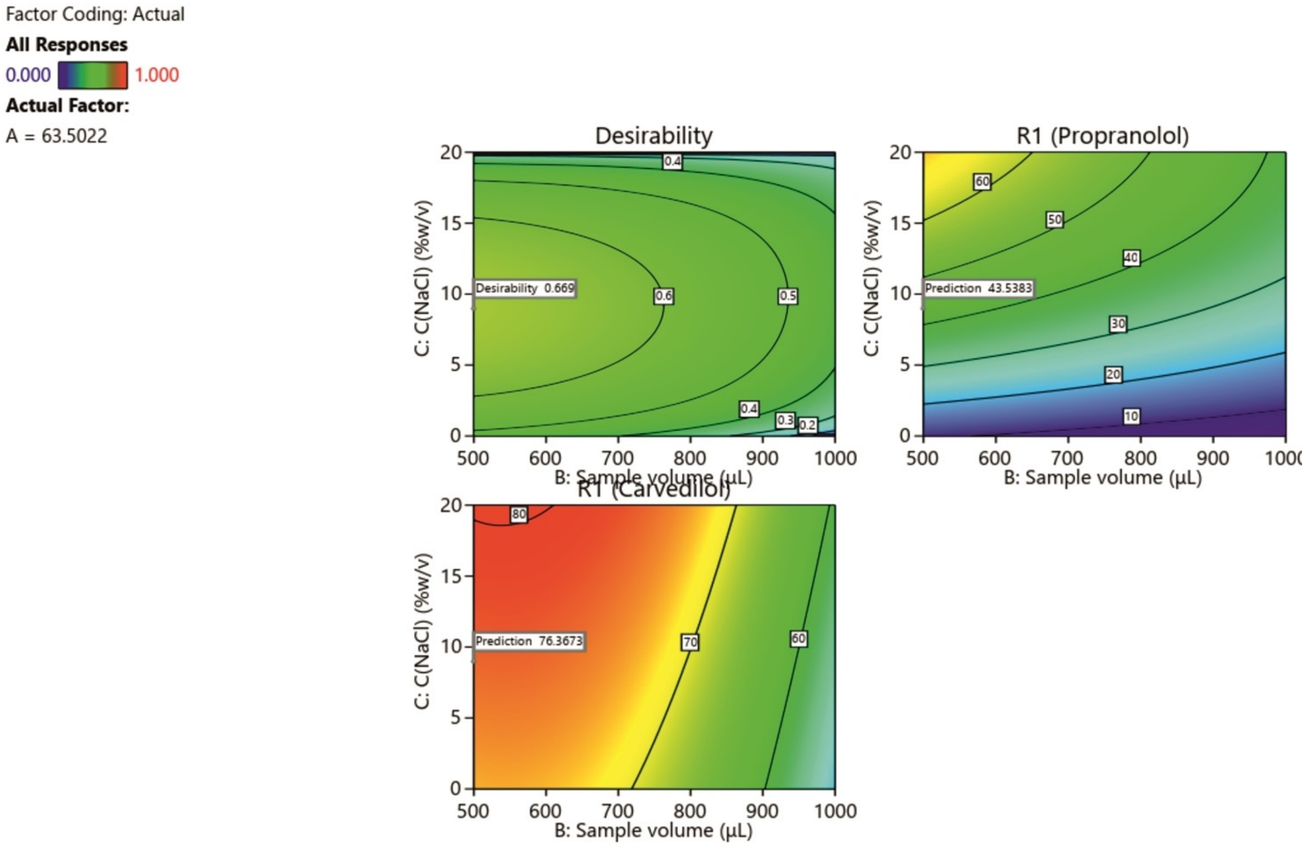

Figure S2. Contour plots of the desirability functions.

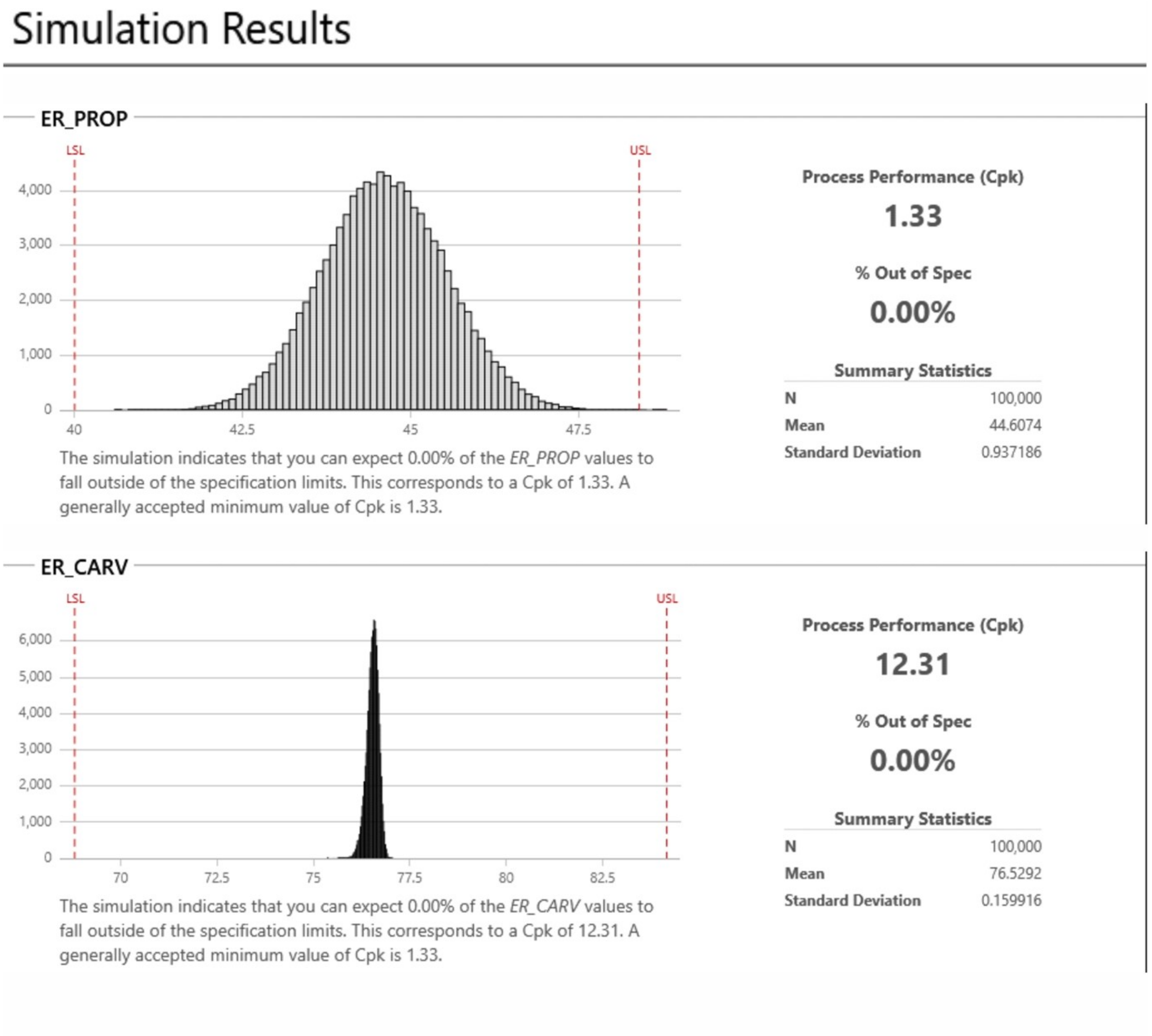

Figure S3. Probabilistic distribution during Monte Carlo simulation studies for both drugs.

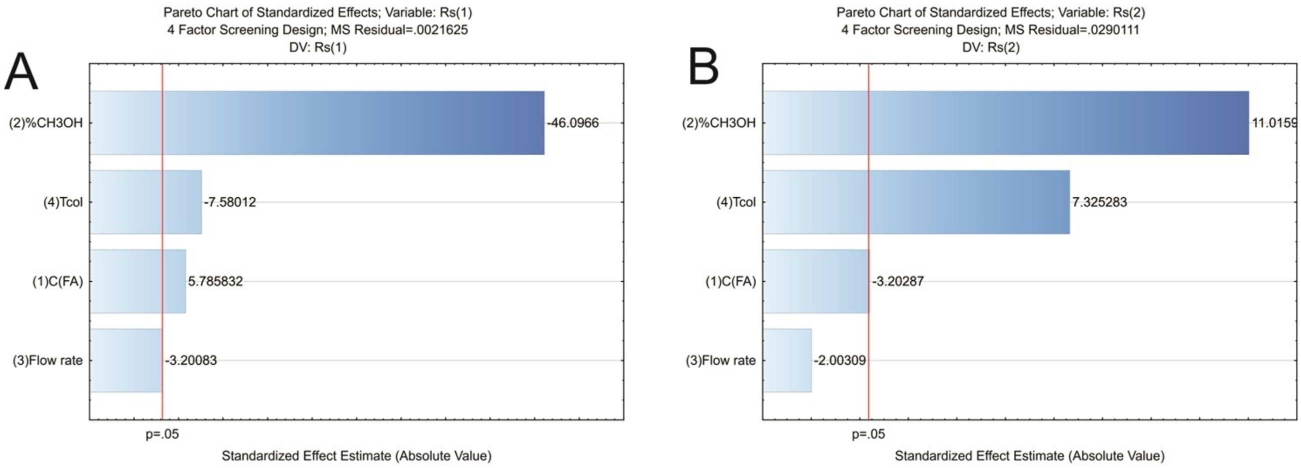

Figure S4. Pareto charts for the robustness test of HPLC parameters. Responses: A) $R_{1(PROP\text{-}CARV)}$, B) $R_{2(CARV\text{-}ISTD)}$.

AGREEprep

Analytical Greenness Metric
for Sample Preparation

13/07/2025 17:32:01

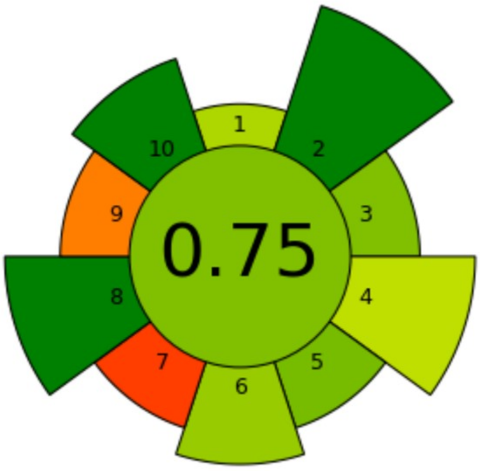

#	Criterion	Score	Weight
1.	**Sample preparation placement**<br>Sample preparation placement: On-line/In situ	0.66	1
2.	**Hazardous materials**<br>Mass [g] or volume [mL] of problematic materials: 0.0	1.0	5
3.	**Sustainability and renewability of materials**<br>> 75% of reagents and materials are sustainable or renewable	0.75	2
4.	**Waste**<br>Mass [g] or volume [mL] of waste: 1	0.63	4
5.	**Size economy of the sample**<br>Mass [g] or volume [mL] of the sample: 0.5	0.77	2
6.	**Sample throughput**<br>Hourly sample throughput: 20	0.71	3
7.	**Integration and automation**<br>No. of sample prep. steps: 4 steps; degree if automation: Manual systems	0.12	2
8.	**Energy consumption**<br>Approximate energy consumption per analysis [W]: 1	1.0	4
9.	**Post-sample preparation configuration for analysis**<br>Liquid chromatography, gas chromatography with quadrupole detection, etc.	0.25	2
10.	**Operator's safety**<br>No. of distinct hazards: No hazards or no exposure	1.0	3

Figure S5. Inputs parameters for AGREEprep tool for method's greenness evaluation.

ComplexMoGAPI

Questions

**SAMPLE PREPARATION**	
**1 - Collection:**	On-line or at-line
**2 - Preservation:**	None
**3 - Transport:**	Required
**4 - Storage:**	Under normal conditions
**5 - Type of method:**	Extraction required
**6 - Scale of extraction:**	Micro-extraction
**7 - Solvents/reagents used:**	Green solvents/reagents used
**8 - Additional treatment:**	Simple treatments (clean up, solvent removal, etc.)
**REAGENT AND SOLVENTS**	
**9 - Amount:**	< 10 mL (< 10 g)
**10 - Health hazard:**	Slightly toxic, slight irritant; NFPA health hazard score = 0 or 1
**11 - Safety hazard:**	Highest NFPA flammability or instability score of 0 or 1. No special hazards
**INSTRUMENTATION**	
**12 - Energy:**	≤1.5 kWh per sample
**13 - Occupational hazard:**	Hermetic sealing of analytical process
**14 - Waste:**	1–10 mL (1–10 g)
**15 - Waste treatment:**	Recycling
**16 - QUANTIFICATION:**	Yes
**YIELD AND CONDITIONS**	
**I - Yield:**	Not applicable
**II - Temperature/time:**	Not applicable
**RELATION TO GREEN ECONOMY**	
**III - Number of rules met:**	5-6
**REAGENTS AND SOLVENTS**	
**IVa - Health hazard:**	Slightly toxic, slight irritant; NFPA health hazard score of 0 or 1
**IVb - Safety hazard:**	Highest NFPA flammability, instability score of 0 or 1. No special hazards
**INSTRUMENTATION**	
**Va - Technical setup:**	Additional setups/semi-advanced instruments used
**Vb - Energy:**	≤1.5 kWh per sample
**Vc - Occupational hazard:**	Hermetization of the analytical process
**WORKUP AND PURIFICATION**	
**VIa - Workup and purification of the end product:**	Not applicable
**VIb - Purity:**	Not applicable
**27. E-FACTOR**	
**This application is for research purposes.**	
**Cite this work as: F.R. Mansour, K.M. Omer, J. Płotka-Wasylka, A total scoring system and software for complex modified GAPI (ComplexMoGAPI) application in the assessment of method greenness, Green Anal. Chem. 10 (2024) 100126.doi:10.1016/j.greeac.2024.100126.**	

Figure S6. Inputs parameters for ComplexMoGAPI tool for method's greenness evaluation.

Table S1. Levels of factors examined by PBD for screening design.

Parameter	Code	Low level (-1)	High level (+1)
Menthol volume (µL)	A	25	250
Sample volume (µL)	B	500	1000
NaCl concentration (% w/v)	C	0	20
Sonication time (min)	D	30	180
Centrifugation speed (rpm)	E	4000	10000
Centrifugation time (min)	F	2	5
Dissolution solvent volume (µL)	G	500	1000
Dummy 1	H	-1	1
Dummy 2	J	-1	1
Dummy 3	K	-1	1
Dummy 4	L	-1	1

Table S2. Plans of experiments generated by PBD for screening design.

Run	A	B	C	D	E	F	G	H	J	K	L	%ER (PROP)	%ER (CARV)
1	137.5	750	10	105	7000	3.5	750	0	0	0	0	32.38	75.39
2	25	500	20	30	10000	5	500	1	1	1	-1	60.39	77.53
3	250	1000	0	180	10000	5	500	-1	-1	1	-1	3.76	55.55
4	25	500	0	30	4000	2	500	-1	-1	-1	-1	6.09	70.62
5	250	500	20	180	4000	5	1000	1	-1	-1	-1	72.27	64.05
6	137.5	750	10	105	7000	3.5	750	0	0	0	0	34.34	75.00
7	25	1000	20	30	10000	5	1000	-1	-1	-1	1	28.42	67.99
8	25	1000	20	180	4000	2	500	1	-1	1	1	32.1	74.60
9	25	1000	0	180	10000	2	1000	1	1	-1	-1	2.89	65.58
10	250	500	20	180	10000	2	500	-1	1	-1	1	55.52	54.59
11	250	500	0	30	10000	2	1000	1	-1	1	1	36.64	64.72
12	250	1000	20	30	4000	2	1000	-1	1	1	-1	45.01	63.04
13	137.5	750	10	105	7000	3.5	750	0	0	0	0	35.03	76.99
14	250	1000	0	30	4000	5	500	1	1	-1	1	4.40	51.73
15	25	500	0	180	4000	5	1000	-1	1	1	1	3.47	63.73

Table S3. ANOVA table for the %ER of PROP.

Source	Sum of Squares	df	Mean Square	F-value	p-value	
**Model**	10210.98	7	1458.71	28.03	< 0.0001	significant
A-Menthol volume	694.39	1	694.39	13.34	0.0033	
B-Sample volume	859.14	1	859.14	16.51	0.0016	
C-C(NaCl)	7136.91	1	7136.91	137.14	< 0.0001	
AC	245.64	1	245.64	4.72	0.0506	
BC	303.44	1	303.44	5.83	0.0326	
A^2	259.34	1	259.34	4.98	0.0454	
C^2	136.56	1	136.56	2.62	0.1312	
**Residual**	624.51	12	52.04			
Lack of Fit	432.06	7	61.72	1.60	0.3118	not significant
Pure Error	192.45	5	38.49			
**Cor Total**	10835.48	19				

[1]degree of freedom

Table S4. ANOVA table for the %ER of CARV.

Source	Sum of Squares	df	Mean Square	F-value	p-value	
**Model**	1454.28	6	242.38	18.03	< 0.0001	significant
A-Menthol volume	0.9000	1	0.9000	0.0669	0.7999	
B-Sample volume	579.12	1	579.12	43.08	< 0.0001	
C-C(NaCl)	132.50	1	132.50	9.86	0.0078	
AB	158.42	1	158.42	11.78	0.0045	
A^2	109.98	1	109.98	8.18	0.0134	
B^2	123.50	1	123.50	9.19	0.0096	
**Residual**	174.76	13	13.44			
Lack of Fit	151.80	8	18.98	4.13	0.0674	not significant
Pure Error	22.96	5	4.59			
**Cor Total**	1629.05	19				

[1]degree of freedom

Table S5. Precision and accuracy of the method for determination of the studied drugs in pooled urine sample.

**Analyte**	**Nominal concentration (ng/mL)**	**Intra-day (*n* = 3)**		**Inter-day (*n* = 3)**	
		**Precision (%RSD)**	**Accuracy (R%)**	**Precision (%RSD)**	**Accuracy (R%)**
Prop	250	5.7	110.2	9.0	107.3
	750	1.9	95.0	5.2	91.1
	2000	3.3	109.5	1.7	89.4
CARV	100	11.0	88.2	2.0	87.2
	500	9.2	98.4	4.4	96.1
	2000	3.5	93.7	1.9	107.5

Table S6. Plackett-Burman design for the evaluation of the robustness of HPLC separation.

Run No	C(FA)	%CH_3OH content	Flow rate (mL/min)	Column temperature (°C)	Dummy1	Dummy2	Dummy3	$R_{(s)1}$[a]	$R_{(s)2}$[b]
3	0.09	51	0.95	24	1	-1	1	2.66	8.37
6	0.11	49	1.05	24	1	-1	-1	4.24	6.52
2	0.11	49	0.95	24	-1	1	1	4.44	6.91
5	0.09	49	1.05	26	-1	-1	1	3.85	7.85
1	0.09	49	0.95	26	1	1	-1	3.94	8.12
7	0.09	51	1.05	24	-1	1	-1	2.60	8.48
8	0.11	51	1.05	26	1	1	1	2.54	8.70
4	0.11	51	0.95	26	-1	-1	-1	2.61	9.13

[a]$R_{(s)1}$: Resolution of PROP-CARV

[b]$R_{(s)2}$: Resolution of CARV-ISTD
